# Illinois State Medical Society

**Published:** 1859-07

**Authors:** 


### ILLINOIS STATE MEDICAL SOCIETY. ANNUAL MEETING AT DECATUR. REPORT OF PROCEEDINGS.

The annual meeting for 1859, of the Illinois State Medical Society, met according to previous appointment, in the city of Decatur, on the 7th of June, and was called to order by the President, Dr. H. A. Johnson, of Chicago.

Dr. Trowbridge, of Decatur, in behalf of the profession and citizens of that city, in a neat and appropriate address, extended a hearty welcome to the Society, and assured its members of the interest which the people and physicians of Macon would continue to feel in its prosperity and usefulness. The roll was called, after which the order of business was suspended to allow of the election of permanent members.

The Society elected the following officers for the ensuing year:

| | |
|---|---|
| *President,* | Dr. David Prince, of Jacksonville. |
| 1*st Vice-President,* | Dr. H. W. Davis, of Paris. |
| 2*d Vice-President,* | Dr. S. T. Trowbridge, of Decatur. |
| *Treasurer,* | Dr. J. W. Freer, of Chicago. |

Drs. Luce, Young, and Chambers were appointed a Committee on Unfinished Business.

Dr. Stormont, of Grand View, presented a report from a committee, appointed at the last meeting of the Society, to memorialize the Legislature to enact a law for the Registration of Births, Marriages, and Deaths in this State.

From the report, it seems that the Legislature did not think the matter was of much importance. The difficulty, we presume, is, that they did not rightly understand the objects to be attained. All experience goes to show that the interests of society may be very much advanced by the provisions prayed for in this memorial.

The committee was continued for another year.

Dr. Young, from Aurora City Medical Association, presented a preamble and resolution, recommending the State Society to appoint a committee, whose duty it should be to use all proper means to secure the appointment of competent medical men to take the census of 1860, in the State of Illinois, and also to recommend what statistics ought to be taken for the benefit of science and health, other than those already provided for by law.

The preamble and resolution was referred to the Committee of Registration of Births, Marriages and Deaths.

The Society then adjourned till afternoon.

AFTERNOON SESSION.

The meeting was called to order at half-past two o'clock, by the President, Dr. Prince.

The report of the Committee on Obstetrics and Diseases of Women, by Dr. W. H. Byford, of Chicago, chairman, was presented, and an abstract read. The report was received, and referred to the Committee on Publication.

The Society proceeded to select a place for the next annual meeting. Jacksonville, Urbana, Chicago and Paris were proposed.

It was finally determined to meet in 1860, at Paris, on the second Tuesday of May.

Dr. Fishback, appointed by the Indiana State Medical Society, as a delegate, to attend the Illinois State Medical Society, was received, and invited to participate in the proceedings of the session.

Dr. E. Powell, of Chicago, chairman of the Committee on Surgery, read an abstract of the report, which was received, and referred to the Publishing Committee.

Dr. J. H. Hollister, of Chicago, presented a report on Nursing Sore Mouth, which was received, and also referred to the Committee on Publication.

The regular committee on Drugs and Medicines made no report.

Dr. Goodwin, of Rockford, a special committee, made no report.

Dr. Freer, of Chicago, also did not report, but was continued by his request.

Dr. Chambers, of Charleston, found that he had left his report on typhoid fever at home, but give a verbal synopsis. He was requested to forward it for publication.

The Committee on Legalizing Dissection made no formal report.

Members of the Committee stated, that the matter was suggested to several members of the Legislature, at the last session, but it was believed that nothing could be done, and, therefore, no formal action was taken.

The committee was continued.

The Society adjourned till Wednesday morning.

SECOND DAY.

Society called to order by the President, Dr. Prince, at half-past eight o'clock.

Minutes of previous day read and adopted.

The Committee of Arrangements reported quite a number of additional delegates and permanent members.

On motion of Dr. Freer, those proposing new members were required to pay the initiation fee for the candidates proposed.

The Committee on Prize Essays reported, that they had determined to award the prize of twenty dollars offered by Dr. J, M. Steele, of Grand View, for the best essay on the Uses of Opium in Inflammatory Diseases, to Dr. A. S. Hudson, of Sterling, Ill., and the prize of fifty dollars offered by the Society, to Dr. Philips, of Dixon.

The subject of this essay was the Influence of Climate on Tuberculosis or Consumption.

Dr. Davis, from Special Committee on Alteration of Blood in Continued Fever, reported in part, and was continued for another year.

Dr. Young extended an invitation to the Society to meet in Aurora in 1861, which was received and placed on file.

On motion, Drs. Brainard and Miller, of Chicago, and Davis, of Paris, were appointed delegates to attend the meeting of the Indiana State Medical Society.

Dr. N. S. Davis, of Chicago, was appointed to deliver the annual address at the next regular meeting of this Society.

Drs. Young, Stormont and Johnson were appointed a committee on prize essays for the ensuing year.

Dr. Whitmire offered twenty dollars as a prize for the best essay on the use of strychnine in the treatment of chronic malarial diseases.

Dr. A. Hard, of Aurora, was appointed a special committee on the medical use of Veratrum Viride.

Dr. E. L. Holmes, of Chicago, was appointed a special committee on Affections of the Eye.

The thanks of the Society were tendered to retiring officers, the Committee on Arrangements, and the citizens of Decatur.

On motion of Dr. Johnson, the Society adjourned, to meet in Paris on the second Tuesday in May, 1860.

On Tuesday, May 7th, the retiring President, Dr. H. A. Johnson, of this city, delivered an address before the Society, on the subject of "Human Dissections, and the interest which the people should feel in the pursuit of anatomical studies."

On motion of Dr. York, it was directed that each member of the Society act as a special committee to procure the publication in his local newspaper of such portions of the address as may in his judgment seem proper.

